# Measuring health-related quality of life in young adolescents: Reliability and validity in the Norwegian version of the Pediatric Quality of Life Inventory™ 4.0 (PedsQL) generic core scales

**DOI:** 10.1186/1477-7525-4-61

**Published:** 2006-09-14

**Authors:** Trude Reinfjell, Trond H Diseth, Marijke Veenstra, Arne Vikan

**Affiliations:** 1 Department of Psychology, Norwegian University of Science and Technology (NTNU), N-7491, Trondheim, Norway; 2 Section of Child and Adolescent Psychiatry, Department of Paediatrics, Rikshospitalet – Radiumhopitalet HF, N-0027 Oslo, Norway; 3 Biostatistics, Rikshospitalet – Radiumhospitalet HF, N-0027, Oslo, Norway

## Abstract

**Background:**

Health-Related Quality of Life (HRQOL) studies concerning children and adolescents are a growing field of research. The Pediatric Quality of Life Inventory (PedsQL™) is considered as a promising HRQOL instrument with the availability of age appropriate versions and parallel forms for both child and parents. The purpose of the current study was to evaluate the psychometric properties of the Norwegian translation of the Pediatric Quality of Life Inventory (PedsQL™) 4.0 generic core scale in a sample of healthy young adolescents.

**Methods:**

A cross-sectional study of 425 healthy young adolescents and 237 of their caregivers participating as a proxy. Reliability was assessed by Cronbach's alpha. Construct validity was assessed using exploratory factor analysis and by exploring the intercorrelations between and among the four PedsQL subscales for adolescents and their parents.

**Results:**

All the self-report scales and proxy-report scales showed satisfactory reliability with Cronbach's alpha varying between 0.77 and 0.88. Factor analysis showed results comparable with the original version, except for the Physical Health scale. On average, monotrait-multimethod correlations were higher than multitrait-multimethod correlations. Sex differences were noted on the emotional functioning subscale, girls reported lower HRQOL than boys.

**Conclusion:**

The Norwegian PedsQL is a valid and reliable generic pediatric health-related Quality of Life measurement that can be recommended for self-reports and proxy-reports for children in the age groups ranging from 13–15 years.

## Background

Mirroring a modern bio-psycho-social orientation toward the concept of health, the development of a multidimensional Health-Related Quality of Life (HRQOL) measurement has been an important concern of research in recent years. It is realized that an instrument measuring HRQOL must consist of the physical, mental, and social health dimensions delineated by the World Health Organization (WHO) [1]. HRQOL studies related to children are a relatively new field of research [2], still there are only a few measures that assess Quality of life outcomes for children and adolescents [3]. Such studies can have considerable significance for understanding children's psychosocial functioning and development like their perception of illness and its effect on their daily life [4,5]. However, the lack of valid and reliable measures for children and adolescents is one significant limitation of current HRQOL research [6].

Issues related to young persons continuous and often rapid developmental change were initially not sufficiently realized [4,7]. A pediatric health-related quality of life (HRQOL) instrument which includes a developmental perspective must for instance show sensitivity to both cognitive and emotional changes throughout the age span. Daily functioning in contexts relevant for children, such as school and community, should also be assessed [8].

Furthermore, a problem of these scales has been low concordances between proxy-and self-reports on HRQOL instruments. This has been observed in studies of children in both pediatric and psychiatric population [9,10]. Concordances tend to be lower for internalizing problems (eg. depression) than for externalizing problems (eg. hyperactivity) [11]. The presence of low concordance between proxy-and self-reports suggests a critical need in pediatric HRQOL measurement for reliable and valid child self-report instruments for the broadest age range possible [9].

The Pediatric Quality of Life Inventory (PedsQL) [9] is considered one of the most promising HRQOL instruments for children and adolescents, integrating generic core scales and disease-specific modules into one measurement system [12].

The instrument includes a broad age range with developmental sensitivity as well as categories for both parents and the young persons themselves. The PedsQL version 4.0 builds on programmatic instrument development research during the past 15 years, beginning with the measurement of pain and functional status [13]. The 4.0 version was designed to measure the core health dimensions delineated by WHO [1], including role (school) functioning [9], and were developed through focus groups and cognitive interviews [6]. The PedsQL 4.0 has been proposed as a valid and reliable generic pediatric HRQOL measurement that can be used for self-reports and proxy-reports in age groups ranging from 2 to 18 years [9], and can also be used in clinical practice, clinical trials, and research, as well as school health settings, and community populations [7,9].

The PedsQL is translated into many European and other international languages, and widely used in research. PedsQL was translated into Norwegian during 2002/2003, at that time no other HRQOL measurements for children were available in Norway. When selecting a HRQOL measure it will be important to examine its psychometric adequacy as well as its ability to tap outcomes of primary interest to a particular investigation [14]. The importance of validating new translations should be emphasized to investigate the acceptability of the psychometric properties for further use in both clinical practice and research. This first validation study of the PedsQL Norwegian version is a pilot study with young adolescents, and is part of a larger study with a broader focus on young adolescent's quality of life and mental health.

The objective of the current paper was to evaluate reliability and validity of the Norwegian translation of the PedsQL™ (version 4.0 generic core scale) in a sample of healthy young adolescents. The focus in the present paper is therefore on the scales that are relevant for adolescents.

## Methods

### Participants

A sample of 440 young adolescents and their parents were recruited through five junior high schools in Norway, three from urban and two from rural areas. A total of 440 questionnaires were distributed and 425 were returned, which gives a response rate of 96.6%.

Self-report forms were completed by 425 adolescents, 235 girls (56%) and 184 boys (44%), six did not report gender. In junior high schools in Norway adolescents between 13 to 15 years of age are separated in three different grades and participants were distributed as follows for 8^th^, 9^th ^and 10^th ^grade; 33%, 33%, 34%, respectively.

Proxy-reports were completed by 237 (56%) caregivers. The proxy-reports were completed by 139 (59%) mothers, by both parents in 69 (29%) of the cases, by 27 (11%) fathers, or by other caregivers such as grandparents 2 (0.8%). For 229 adolescents both adolescent self-report and parent proxy-report on the PedsQL were available. Information about non-response in the sample of adolescents as well as the sample of parents was not available, because of the anonymity required. Sociodemographic characteristics of the sample are given in Table 1. The Data Inspectorate and the Regional Committee for Medical Research Ethics approved the study. Written parental informed consent and child assent were obtained.

**Table 1:** Sociodemographic characteristics of 419 adolescents and their parents

**Adolescents**	**N**	**%**
Total sample	419	
Girls	235	56.1
Boys	184	43.9
School grade:		
8^th ^grade	140	32.9
9^th ^grade	142	33.4
10^th ^grade	143	33.7
		
**Parental education and economy**		
Mothers	216	
Mothers education:		
Elementary school	10	4.7
Highschool graduate	51	23.6
Post high school	155	71.7
		
Fathers	110	
Fathers education:		
Elementary school	5	4.4
Highschool graduate	25	22.8
Post high school	80	72.8
		
Economy	235	
Very satisfying	26	11.1
Good	198	84.2
Poor	11	4.7

### Measures

The 23-item PedsQL, version 4.0 Generic Core Scales, can be grouped into 4 domains of HRQOL: 1) Physical Functioning (8 items), 2) Emotional Functioning (5 items), 3) Social Functioning (5 items) and 4) School Functioning (5 items). These scales are feasible for child self-report including ages 5 to 7, 8 to 12 and 13 to 18 years. Parent proxy-report includes ages 2 to 4, 5 to 7, 8 to 12 and 13 to 18, and assesses parent's perceptions of their child's HRQOL.

The items for self-report and proxy-report are essentially identical, differing in developmentally appropriate language, and first or third person tense. The instructions ask how much of a problem each item has been during the past 1 month. A 5-point response scale is utilized across child self-report for ages 8 – 18 and parent proxy-report (0 = never a problem; 1= almost never a problem; 2 = sometimes a problem; 3 = often a problem; 4 = almost always a problem). Subjects are requested to rate how much problems they experienced during the past month with health (eg. "I hurt or ache"), activities (eg. "It's hard for me to run"), or feelings (eg. "I feel afraid or scared").

Items are reverse-scored and linearly transformed to a 0 to 100 scale (0 = 100, 1 = 75, 2 = 50, 3 = 25, 4 = 0), so that higher scores indicate better HRQOL. Scale scores are computed as the sum of the items divided by the number of items answered (this accounts for missing data). In addition to the four subscales, two summary scores can be computed. Physical Health Summary score (8 items) is the same as the Physical Functioning subscale, and Psychosocial Health Summary score (15 items) is computed as the sum of the items divided by the number of items answered in the Emotional, Social, and School Functioning subscales.

The translation and linguistic validation of the PedsQL questionnaire followed recommended guidelines [15,16]. Two independent forward translations were conducted by a psychiatrist and a clinical psychologist, the translators discussed semantic and conceptual discrepancies and finally developed a consensus forward translation. The translation of the first reconciled forward version of the PedsQL questionnaire back into the source language was done by a skilled english speaking person with experience from living in English speaking countries.

In a following pilot-project, the questionnaire was administrated to 10 children, 12 adolescents and 23 parents to test the interpretation and understanding of items and response ratings. Cognitive interview techniques [15] were used to obtain feedback about the interpretation and understanding of items and respons ratings. The questionnaires were then revised in response to feedback from children and parents. A written report was sent to Varni for further review. The relevant changes in the translation process were reviewed and authorised by Varni. In addition to the PedsQL 4.0, the questionnaires included information about children's socio-demographic characteristics.

### Procedure

Local junior high schools were contacted and teachers distributed written consent forms that the adolescents presented to their parents. Each pupil received an envelope, which contained information and a questionnaire for their parents. Parents were asked to return the completed questionnaire in a pre-stamped envelope. The participants could further contact the researchers to obtain additional information. Approvals signed by the parents and returned to the teacher, confirmed that the adolescent had permission to participate.

The self-report instruments were administrated and completed in the classrooms. Children were given verbal and written information before completing questionnaires in class, under the supervision of a research assistant.

### Statistical analysis

Scale internal consistency reliability was determined by calculating Cronbach's alpha coefficient [17]. Scales with reliabilities equal to or greater than 0.70 were considered satisfactory and are also recommended for comparing patient groups [18,19].

We used exploratory factor analysis to examine the structure of relationships between the items of the PedsQL™ 4.0 and to compare the factor structure in the present study with the structure reported for the original PedsQL™ [9]. Regarding Varni's results where the school functioning items loaded on two separate factors, we expected to find a five factor structure. To extract the factors we applied Principal Component Analysis, with oblique rotation (Direct Oblimin). Factors with an eigenvalue less than 1 were disregarded.

Validity was further examined by exploring the intercorrelations between and among the four PedsQL Subscales [20]. To strengthen faith in the validity of the PedsQL version 4.0, multitrait-monomethod correlations (eg. correlations among subscales within self-report and proxy-report) should be lower than monotrait-multimethod correlations (eg. concordance between self-report and proxy-report for the same subscale). Correlations are designated as small (0.10–0.29), medium (0.30–0.49), and large (=0.50) [21]. Given shared method variance [18] and that the PedsQL items were developed to measure an integrated multidimensional construct (pediatric HRQOL), it was expected that heterotrait-monomethod correlations among the Subscales would be medium to large (0.30–0.50). Proxy/child concordance for the same subscale was furthermore expected to demonstrate medium to large effect sizes.

Based on previous literature [9] it was anticipated that the Physical Functioning Subscale would demonstrate the largest concordance, and heterotrait-heteromethod concordance was expected to be small. In addition, we calculated intraclass correlation coefficients (ICC) to assess parent and child convergence on the PedsQL subscales. ICC takes into account not only the correlation but also differences in intercept and slope between replicant ratings [22]. Paired t-test were used to assess the extend to which adolescents or proxies systematically scored lower on the subscales of the PedsQL. As a measure of the minimally important difference in scores, we calculated the standardized response mean, a distribution-based approach that compares temporal change by the standard deviation of change [21]. Standardized response mean of 0.2–0.5, 0.5–0.8, and >0.8 are regarded as small, moderate, and large, respectively. Gender differences in the self-report scales were analysed with two-sample t-test. For all analyses, we used SPSS statistical software version 12.0 (SPSS Inc., Chicago, III, USA) and a critical value (α) of 5%.

## Results

### Scale-level analysis

Mean scale scores, percentage of scores at the floor and ceiling and Cronbach's alpha are shown in Table 2. All the self-report scales and proxy-report scales exceeded the minimum reliability standard of 0.70. No floor effects were found on self or proxy-report for this healthy sample of adolescence. Ceiling effects existed and ranged from minimal (eg. 2.6% and 3.4% for self and proxy-report, respectively for Total score) to moderate (eg. 26.5% and 24.2% for self and proxy-report for Physical Functioning). The largest effect was found for Social Functioning (43% and 46% for self and proxy-report). Table 2 gives information about scale descriptives and internal consistency reliability for the PedsQL 4.0.

**Table 2:** Scale Descriptives and Internal Consistency Reliability for PedsQL 4.0

Scale	Items	N	Mean	SD	Percentage floor	Percentage ceiling	Cronbach's alpha
**Adolescent self-report**							
Total score	23	414	85.29	11.11	0	2.6	.84
Physical health	8	422	91.12	10.35	0	26.5	.78
Psychosocial health	15	416	82.16	12.50	0	3.1	.82
Emotional functioning	5	424	77.15	17.32	0	10.4	.79
Social functioning	5	424	88.12	13.11	0	43.6	.80
School functioning	5	424	78.02	15.47	0	8.3	.73
**Parent proxy-report**							
Total score	23	232	86.10	10.20	0	3.4	.77
Physical health	8	236	88.83	11.76	0	24.2	.80
Psychosocial health	15	234	84.66	10.92	0	4.3	.88
Emotional functioning	5	238	79.98	14.13	0	12.7	.78
Social functioning	5	238	88.05	13.37	0	46.0	.82
School functioning	5	238	88.97	12.37	0	13.5	.75

Further, for all 23 items, item means for self-report ranged from 67.9 to 99.9 with 12 of 23 items falling within a 10-point range. Item means for proxy report ranged from 67.8 to 98.9, with 13 items falling within a 10-point range. Two items from the Physical health scale have a relatively small standard deviation namely: 1.2 (item 5) and 9.6 (item 1) for the self-report, 8.2 (item 5) and 8.8 (item 1) for the proxy report. The remaining standard deviations ranged from 13.3 to 25.8 for self-report items and 14.8 to 23.7 for proxy-report items.

### Construct validity

#### Adolescent-parent report

Monotrait-multimethod correlations are all statistically significant but generally modest. Table 3 shows the intercorrelations between and among the four subscales of the PedsQL.

**Table 3:** Intercorrelations between and among PedsQL subscales

	Adolescent self-report				Parent proxy-report		
**Adolescent self-report**	1	2	3	4	5	6	7
1 Physical functioning							
2 Emotional functioning	**0,65**						
3 Social functioning	**0,66**	**0,61**					
4 School functioning	**0,56**	**0,54**	**0,46**				
**Parent proxy-report**							
5 Physical functioning	0,35	*0,12*^ns^	*0,21*	*0,35*			
6 Emotional functioning	0,25	0,22	*0,29*	*0,30*	**0,52**		
7 Social functioning	*0,20*	*0,12*^ns^	0,28	*0,25*	**0,51**	**0,50**	
8 School functioning	*0,17*	*0,15*	*0,19*	0,42	**0,53**	**0,54**	**0,49**

Notes: N = 229; NS = Not significant at 5% level; Multitrait-monomethod correlations are in bold; monotrait-multimethod correlations are underlined; multitrait-multimethod correlations are italicised.

For the subscale School Functioning we found moderate (>0.40) intercorrelations between adolescents and parents. All multitrait-multimethod correlations were lower than the monotrait-multimethod correlations. However, some of the multitrait-multimethod correlations are higher than the convergent correlations of the other three subscales, in particular for Emotional functioning and Social functioning. The average convergent correlation is 0.31 and the average off-diagonal correlation is 0.22. This indicates that on average the monotrait-multimethod correlations are higher than the multitrait-multimethod correlations. The intraclass-correlation (ICC) was relatively low for all scales, indicating poor to fair (<0.40) child-proxy agreement for all scales but one. Moderate agreement (ICC = 0.41) was found for the sub scale measuring School functioning. Lowest agreement was found for the emotional functioning scale (ICC = 0.21). The results of the paired t-tests suggested that parents scores were systematically higher than that of adolescents for Emotional functioning (t = 2.32; df = 228; p = 0.02) and School functioning (t = -5.28; df = 228; p < 0.001). Conversely, parents reported lower on the subscales for Physical functioning (t = 2.9; df = 233; p = 0.004) and Psychosocial health scale (t = -2.7; df = 231; p = 0.007). The scores on the Social Functioning scale did not yield statistically significant differences between parents and adolescents (t = 1; df = 228; p = 0.268). Only the difference found for School Functioning corresponded to a small effect size (0.35), the other differences between parents and adolescents all have effect sizes below 0.20.

#### Gender differences

A statistically significant gender differences was found on the emotional subscale, with girls on average scoring lower than boys (t = 4,79; df = 416, p < 0.001). However, the mean score for girls (73.92 and sd = 17.53) as well as for boys (81.85 and sd = 15.83) were at the high end of the scale. No statistically significant gender differences were found for the remaining scales.

### Factor analysis

The results of the factor analysis for self-report and proxy-report are shown in table 4 and 5.

**Table 4:** PedsQL 4.0 Norwegian version Factor Loadings for Adolescents Self-Report

Scale/Item	Factor 1	Factor 2	Factor 3	Factor 4	Factor 5
**Physical Functioning**					
1. Hard to walk more than one block	,079	**,702**	-,356	,086	,299
2. Hard to run	,327	**,887**	-,359	,145	,270
3. Hard to do sports or exercises	,375	**,851**	-,322	,142	,240
4. Hard to lift something heavy	,251	**,568**	-,484	,052	,363
5. Hard to take bath or shower	-,036	,039	,041	**,509**	-,195
6. Hard to do chores around house	**,497**	,255	-,363	-,008	,421
7. Hurth or arche	**,562**	,460	-,441	,229	,436
8. Low energy	,531	**,595**	-,474	,131	,371
**Emotional Functioning**					
1. Feel afraid or scared	**,723**	,344	-,479	,183	,323
2. Feel sad or blue	**,799**	,419	-,385	,092	,387
3. Feel angry	**,742**	,210	-,363	,027	,418
4. Trouble sleeping	**,553**	,301	-,249	,182	,305
5. Worry about what will happen	**,641**	,387	-,492	,035	,489
**Social Functioning**					
1. Trouble getting along w/peers	,500	,349	**-,730**	,032	,219
2. Other kids not wanting to be friend	,481	,369	**-,770**	,164	,213
3. Teased	,450	,299	**-,583**	,163	,161
4. Doing things other peers do	,201	,401	**-,772**	,061	,341
5. Hard to keep up when play with others	,247	,400	**-,799**	,019	,412
**School Functioning**					
1. Hard to concentrate	,377	,326	-,341	,129	**,825**
2. Forget things	,435	,335	-,330	,024	**,799**
3. Trouble keeping up with schoolwork	,418	,387	-,317	,100	**,825**
4. Miss school – not well	,462	,287	-,161	**,571**	,286
5. Miss school – doctor appointment	,109	,105	-,163	**,791**	,251

Eigenvalue cutoff: 1.0; Total Variance Explained for Adolescents Self-Report: 57%; Bold = highest factor loading for each item.

**Table 5:** PedsQL 4.0 Norwegian version Factor Loadings for Parent Proxy-Report

Scale/Item	Factor 1	Factor 2	Factor 3	Factor 4	Factor 5
**Physical Functioning**					
1. Hard to walk more than one block	,151	**,808**	-,143	,190	-,263
2. Hard to run	,322	**,816**	-,364	,305	-,149
3. Hard to do sports or exercises	,302	**,812**	-,392	,326	-,196
4. Hard to lift something heavy	,230	**,774**	-,260	,225	-,253
5. Hard to take bath or shower	,003	**,687**	-,152	,196	-,210
6. Hard to do chores around house	,052	,309	-,272	,291	**-,738**
7. Hurth or arche	,491	,339	-,202	,279	**-,664**
8. Low energy	,428	,421	-,426	,384	**-,692**
**Emotional Functioning**					
1. Feel afraid or scared	**,775**	,187	-,281	,294	-,263
2. Feel sad or blue	**,716**	,338	-,471	,367	-,411
3. Feel angry	**,597**	,166	-,293	,420	-,387
4. Trouble sleeping	**,680**	,158	-,159	,180	-,112
5. Worry about what will happen	**,715**	,344	-,432	,391	-,141
**Social Functioning**					
1. Trouble getting along w/peers	,327	,316	**-,833**	,279	-,259
2. Other kids not wanting to be friend	,337	,231	**-,905**	,358	-,118
3. Teased	,237	,277	**-,776**	,359	-,169
4. Doing things other peers do	,241	,530	**-,618**	,462	-,013
5. Hard to keep up when play with others	,170	,359	-,545	**,618**	,026
**School Functioning**					
1. Hard to concentrate	,321	,278	-,296	**,852**	-,222
2. Forget things	,222	,267	-,222	**,761**	-,370
3. Trouble keeping up with schoolwork	,363	,207	-,369	**,841**	-,214
4. Miss school – not well	,408	,250	-,108	,352	**-,661**
5. Miss school – doctor appointment	**,386**	,147	-,135	,348	-,263

Eigenvalue cutoff: 1.0; Total Variance Explained for Proxy-Report: 60%; Bold = highest factor loading for each item.

An eigenvalue cutoff of 1.0 resulted in a five factor solution for self-report and proxy-report, accounting for 56 % and 61 % of the variance. The school functioning items split into two different factors, like the originally version. For physical functioning, item 5 ("hard to take bath or shower"), item 6 ("hard to do chores around the house") and item 7 ("hurth or arche") split into different factors. The items related to emotional and social functioning are consistent with the original PedsQL™ version [23].

### Discussion

This article describes the psychometric properties of the Norwegian translation of the PedsQL™ 4.0 generic core scale in a healthy sample of young adolescents and their caregivers. The results from the present study resemble the findings of the original PedsQL™ [9] and the UK-English version [24] and as such confirm that the instrument can be used for self-reports and proxy-reports in school health settings and community populations.

### Reliability

Internal consistency was satisfactory with Cronbach's alph coefficient >0.70 for all four subscales. No floor effects were found for any of the scales. The presence of ceiling effects in the present study may be expected in generic HRQOL instruments, because they are made to be applicable to a wide range of populations [24]. This could be a sample specific phenomenon, and should be further explored through the administration of PedsQL™ to children with different health issues including those children and adolescents experiencing acute health problems.

Regarding single item descriptives, it is interesting to note the low standard deviation for two items from the Physical health scale. These results are challenging the requirements of equivalent item means and variance. However, this finding may be typical for the way PedsQL behaves in a healthy sample.

### Validity

Our results showed that on average the monotrait-multimethod correlations are higher than the multitrait-multimethod correlations. This high multitrait-multimethod correlations indicate that the different traits measured in the four subscales show considerable overlap. For example, three items in the physical functioning scale ("hard to take bath or shower", "hard to do chores around the house", and "hurt or ache") are loading on another factor than the other physical functioning items. This could be more related to a fatigue component, which seems more relevant for a chronically ill patient population than healthy adolescents. A confirmatory factor analysis could provide further insight in the degree of overlap between items hypothesized to measure different constructs, and also in the equivalence of factor loadings on the items within a single factor.

The adolescent-parent agreement did not exceed the preferred intra-class correlation of 0.40, except for the scale measuring School function. Lack of agreement between parents and children may result from differences in perception of the same situation, and also differences in interpretation of different items [11], or may be due to the young adolescents becoming more independent from the parents. As opposed to some previous research [25], our findings did not find higher agreement between parents and adolescents regarding physical problems. Parents rated the physical function scale lower than their children's reports. Further, a recent study found that proxy and self-report correlation was higher for children with health problems than for healthy children [24]. Parents and children may be more likely to share information about an issue if it is perceived as a problem [24]. However, the strength of this agreement has also been challenged in research on children with Cystic Fibrosis [8].

Another explanation for the low concordance between adolescents and parents regarding physical functioning can be seen in the factor analysis (table 4 and 5) which indicated that items concerning physical functioning (5, 6, 7) were rather diffuse components related to physical as well as emotional domains, and therefore difficult to distinguish, something that could further influence both adolescents and parents ratings. Children reported lower HRQOL on the emotional scale compared with their parents, and corresponds to the previous research of Modi & Quittner [8]. Young children may have difficulty expressing their emotions directly to their parents, another factor could be the likeliness that proxy-report reflect parental anxiety about their child [24]. This aspect should be further investigated in different patient populations, and confirms the need to measure both child and parent perspectives when evaluating HRQOL. Clinically, those discrepancies give a potential for interventions emphasizing the children's subjective ratings, as well as their parents [8,11].

Regarding gender differences, we found that girls reported lower levels of emotional functioning than boys. This is consistent with previous research regarding gender differences in emotional health [26-28]. The gender differences would seem to reflect a genuine disparity between boys and girls and therefore gives further evidence for the validity of PedsQL™ as a sensitive measure of the emotional functioning of children and adolescents [24].

The result of the factor analysis resembles Varni's five-factor structure in the original PedsQL™ version, except for some items. Like the results of Varni *et al*. [9] two of the five items (4 and 5) related to school functioning were loading to another factor. A natural explanation for this could be that the three first items related to school functioning (eg. "hard to concentrate", "forget things", "trouble keeping up with schoolwork") are more likely to have a cognitive component, while the others are more related to physical aspects (eg. "miss school because not feeling well", "miss school because of doctor appointment").

All items related to social functioning had a clear factor loading, as well as the items related to emotional functioning. The physical items seem to split into three factor loadings (see Table 4). Item 1 ("hard to walk more than a block"), item 2 ("hard to run"), item 3 ("hard to do sports or exercises"), item 4 ("hard to lift something heavy") and item 8 ("low energy") are all loading on factor 2. Further, item 6 ("hard to do chores around the house", item 7 ("hurth or arche") on factor 1. Item 5 ("hard to take bath or shower") on factor 4. In the results of Varni *et al*. [9] the loading for the four first items for the physical functioning scale is similar to our results. The factor loadings for the proxy-report also indicate that the physical factor loadings seem to have the same pattern, most of the factor loadings are similar to the child self-report. It should be pointed that comparisons to the factor structure obtained in the original PedsQL™ publication may be restricted and less comparable due to the restricted age range in this present study. The restricted age range, with a healthy population, may attenuate the variability achieved. The results from the factor analysis regarding item 5, 6 and 7 in the Physical Functioning scale, as well as items 4 and 5 in the School Function scale may be typically for healthy samples, the factor structure should therefore be reinvestigated in clinical samples.

### Limitations

Concerning the Norwegian PedsQL™ 4.0 validation study, the present findings have several potential limitations. Test-retest reliability and responsiveness are not reported. Information on non-participants was not available, something that can limit generalizability. In the American validation study, the PedsQL differentiated HRQOL between healthy children and children with acute or chronic health conditions. This will also be an important future goal to investigate for the Norwegian PedsQL version, and is something the authors are taking into consideration. In this study the age range utilized was quite restricted. Regarding developmental aspects, further research should investigate the Norwegian PedsQL versions' psychometric properties concerning the upper-age range which the adolescent PedsQL was made for, as well as younger age-groups.

## Conclusion

The PedsQL Norwegian version is generally a valid and reliable instrument, replicating some of the earlier findings for the originally version. The Norwegian PedsQL™ 4.0 version will be a valuable tool for assessing the HRQOL of young adolescents in Norway.

The imperfect concordance observed between self-and proxy-reports supports the need to measure the perspectives of child and parent in evaluating pediatric HRQOL [9,29]. It would be important emphasizing the clinically usefulness regarding child-parent discrepancies still when challenging the validity of measures.

## Competing interests

The author(s) declare that they have no competing interests.

## Authors' contributions

TR made contribution to the study design, data collection, statistical analysis, interpretation of data and the drafting of the paper. THD contributed to the study design, interpretation of the data, drafting and revising the manuscript. MV contributed to the statistical analysis, interpretation of the data and manuscript drafting. AV has contributed the interpretation of the data and manuscript drafting. All authors read and approved the final manuscript.
